# 

**Published:** 1901-10-12

**Authors:** 


### NOTICE TO CORRESPONDENTS.

All MSS., Letters, Books for Review, and other matters intended for the Editor should be addressed The Editor, "The Hospital," The Hospital Building, 28 & 29 Southampton Street, Strand, W.C.

The Editor cannot undertake to return rejected MS., even when accompanied by stamped directed envelope.

**Notice to Advertisers and Subscribers.**

All Advertisements, Orders for Copies of The Hospital, and Business Communications should be addressed to **THE MANAGER (Not to the Editor),** The Hospital, 28 & 29 Southampton Street, Strand, London, W.C.

The Cost of Subscription, post free, is as follows:—Per week, 2½d.; per quarter, 2s. 8d.; per half-year, 5s. 3d.; per annum, 10s. 6d. Foreign:—Per annum, 15s. 2d., payable, in all cases, in advance.

**NOTICE.—Vol. XXX. of "The Hospital" (April 1901, to September 1901), in handsome cloth binding will shortly be on sale at the Office of this Journal, price, 7s. 6d. Cases for binding the Numbers contained in this Volume 1s. 6d. Index to Vol. XXX., 2½d., post free.**

**The Monthly Part for October is now ready. Price 6d., by post 8d.**

### BOOKS RECEIVED.

John Dicks.

"The Romance of Old London." By Edwin Oliver. With illustrations by W. Amor Fenn and Harry Evans.

Simpkin, Marshall, Hamilton, Kent, and Co.

"Home Exercises for Spinal Curvature." By Richard Limberg.

The Australian Book Company.

"Our Army in South Africa."

Society for Promoting Christian Knowledge.

"The Asylum House."
"Visiting Day at the Asylum."
"Friendly Talk with a New Patient."
"An Address to Asylum Attendants."
"Treatment of the Insane." By the Rev. Henry Hawkins, M.A.

### Scraps and Gleanings.

The number of passengers carried this season by the New Palace steamers is 326,950, being an excess over the previous year of 25,000.

A donation of £100 has been promised by the Leeds Fireclay Company to the fund for the new Building and Endowment Fund o f the Parkes Museum.

During the past year the increase in the number of patients at the Sheffield Royal Infirmary was 3,323, while the increase in expenditure was £3,376.

The money collected in the streets of Scarborough on Hospital Saturday this year, in aid of the Scarborough Hospital and Dispensary, amounted to £254. Last year the amount collected was £280.

By the will of the late Mr. Robert Bayly a sum of £2,000 is left in trust to pay three-fourths of the income to the South Devon and Cornwall Hospital, and one-fourth to the Female Orphan Asylum, Plymouth.

During the present year 400 patients will be received at the Derbyshire Royal Infirmary Convalescent Home. During the first year of its existence 300 patients were received. The income of the home is only £500, whereas £700 is needed to place the finances in a satisfactory condition.

The new ward at the Lytham Cottage Hospital was recently opened by Lady Drummond. A brass tablet in the ward records the fact that the ward has been erected by public subscription in memory of the late Luke Fisher, M.D., who for many years acted as honorary medical superintendent and honorary secretary of the hospital.

The twenty-fifth annual report of the Stockton Hospital Governors shows that the total cost of the institution up to date has been £15,938, and the cost per patient per day to be 4s. 1¾d., against 4s. 6½d. in 1882. The number of in-patients has increased since 1882 from 148 to 789, and the average number of days' residence in the hospital has been reduced during this period from 29 to 17¾ days per patient.

Further subscriptions are much needed to enable the cottage home scheme of the Prince Christian Victor Memorial Fund to be carried out. The idea is to establish one or more cottage homes for each regiment, where the disabled soldiers of that regiment may live rent free. The cost of endowment of such a cottage in perpetuity is £400 to £500. The Soldiers' and Sailors' Help Society will look after the occupants and supplement their pensions when necessary.

### The Student of Medicine.

### APPOINTMENTS.

**J. Johnston Abraham**, Sen. Mod. M.B., B.Ch., B.A.O.Dub., L.M.Rotunda, appointed House Surgeon to the West London Hospital. **J. C. M. Bailey**, M.B.Lond., M.R.C.S., L.R.C.P.Lond., appointed House Physician to the West London Hospital. **Edward Eldridge Blomfield**, M.D., B.S.Lond., M R.C.S., L.R.C.P.Lond., appointed Examiner in Medical Anatomy and Lecturer on Materia Medica at the Otago University, New Zealand. **Robert Eatough**, M.D., C.M.Aberd., appointed Medical Officer and Public Vaccinator for the Twelfth District of the Ashton-under-Lyne Union. **Walter Garstong**, M.B., Ch.B.Vict., appointed House Surgeon to the Royal Lancaster Infirmary. **F. L. Harper**, M.R.C.S.Eng., appointed Certifying Factory Surgeon for the Brixworth Rural District, Northampton. **Miss E. McCall**, M.B., B.S.Glasg., appointed Resident Assistant Medical Officer of the Birkenhead Union. **G. E. May**, M.R.C.S.Eng., L.R.C.P.Lond., appointed Certifying Factory Surgeon for the Ware District of Hertfordshire. **George Robertson**, L.R.C.P. L.R.C.S.Edin., appointed Certifying Factory Surgeon for the Kirkpatrick Durham District of Kirkcudbrightshire. **John Ryan**, L.R.C.P., L.R.C.S.Irel., appointed Certifying Factory Sugeon for the Northleach District of Gloucestershire. **A. E. Shepherd**, L.R C.P., L.R.C S.Edin., appointed Honorary Surgeon to the Adelaide Hospital. **O. C. G. Shields** M.B., appointed Medical Officer of Health for Yea, Victoria. **J. H. Sleeman**, M.B., appointed Officer of Health for Ballin, E., Victoria. **E. Somerville**, M.B., Ch.B.Edin., appointed District Medical Officer of the Leek Union. **C. B. Snow**, B.A., B.Sc., M.B., Ch.B.Edin., appointed House-Physician to the David Lewis Northern Hospital, Liverpool. **G. Arbour Stephens**, M.D., B.S.Lond., appointed Honorary Physician to the Royal Cambrian Institution for Deaf and Dumb. **E. S. Stokes**, M.B., Ch.M.Syd., appointed Assistant Medical Officer of Health for the Sydney Metropolitan Combined District. **D'Arcy Sugden**, L.R.C.P.Lond., M.R.C.S.Eng., appointed District Medical Officer of the Totnes Union. **R. G. C. Thornton**, M.B., B.Ch.R.U.I., appointed District Medical Officer of the Wellington (Salop) Union. **E. T. Wayne**, M.D.Camb., L.R.C.P.Lond., M.R.C.S.Eng., appointed to the Hospital at St. George, Queensland.

### Medical Appointments.

MEDICAL SUPERINTENDENT,

